# Functional Genomics Approaches to Studying Symbioses between Legumes and Nitrogen-Fixing Rhizobia

**DOI:** 10.3390/ht7020015

**Published:** 2018-05-18

**Authors:** Martina Lardi, Gabriella Pessi

**Affiliations:** Department of Plant and Microbial Biology, University of Zurich, CH-8057 Zurich, Switzerland; martina.lardi@uzh.ch

**Keywords:** alpha-rhizobia, beta-rhizobia, symbiosis, transcriptomics, proteomics, metabolomics, flavonoids, root exudates, root nodule

## Abstract

Biological nitrogen fixation gives legumes a pronounced growth advantage in nitrogen-deprived soils and is of considerable ecological and economic interest. In exchange for reduced atmospheric nitrogen, typically given to the plant in the form of amides or ureides, the legume provides nitrogen-fixing rhizobia with nutrients and highly specialised root structures called nodules. To elucidate the molecular basis underlying physiological adaptations on a genome-wide scale, functional genomics approaches, such as transcriptomics, proteomics, and metabolomics, have been used. This review presents an overview of the different functional genomics approaches that have been performed on rhizobial symbiosis, with a focus on studies investigating the molecular mechanisms used by the bacterial partner to interact with the legume. While rhizobia belonging to the alpha-proteobacterial group (alpha-rhizobia) have been well studied, few studies to date have investigated this process in beta-proteobacteria (beta-rhizobia).

## 1. Introduction

Nitrogen fixation in agricultural systems is of enormous agricultural importance, as it increases in situ the fixed nitrogen content of soil and can replace expensive and harmful chemical fertilizers [1]. For over a hundred years, all of the described symbiotic relationships between legumes and nitrogen-fixing rhizobia were confined to the alpha-proteobacteria (alpha-rhizobia) group, which includes *Bradyrhizobium*, *Mesorhizobium*, *Methylobacterium*, *Rhizobium*, and *Sinorhizobium* [2]. However, in 2001, beta-proteobacteria belonging to the genera *Burkholderia* and *Cupriavidus* were first described as being able to nodulate legumes and fix nitrogen [3]. These so-called beta-rhizobia have been isolated mainly from *Mimosa* species (subfamily Mimosoideae) from different continents, but also from papilionoid legumes [4,5,6,7,8,9,10,11,12]. The rapid increase in the number of described legume-nodulating rhizobial strains over the last 20 years [13,14,15] can be attributed to advances in next-generation sequencing (NGS) technologies, which have allowed the sequencing and de novo assembly of the complete genomes of previously unsequenced and newly discovered bacterial species [16].

Rhizobia are able to switch from their free-living state into an N_2_-fixing symbiotic state inside root and stem nodules of certain legumes [17,18,19]. The sequential molecular mechanisms that lead to the infection of the legume and to the differentiation of the bacteria into bacteroids in a mature functional nodule have been studied in detail for alpha-rhizobia [20,21,22,23,24,25,26,27,28,29]. The sequencing and public availability of the complete genomes of several rhizobia and legumes have allowed researchers to develop and apply functional genomics approaches to comprehensively understand how rhizobia reorganize and adapt to new environments, such as the root nodule [30,31,32,33,34]. Such approaches include transcriptomics and proteomics, which report changes in transcript and protein profiles, respectively. In addition to transcriptomics and proteomics, analysis of metabolites can be carried out to allow for integration of the complex interactions between genotype and phenotype.

**Figure 1:**
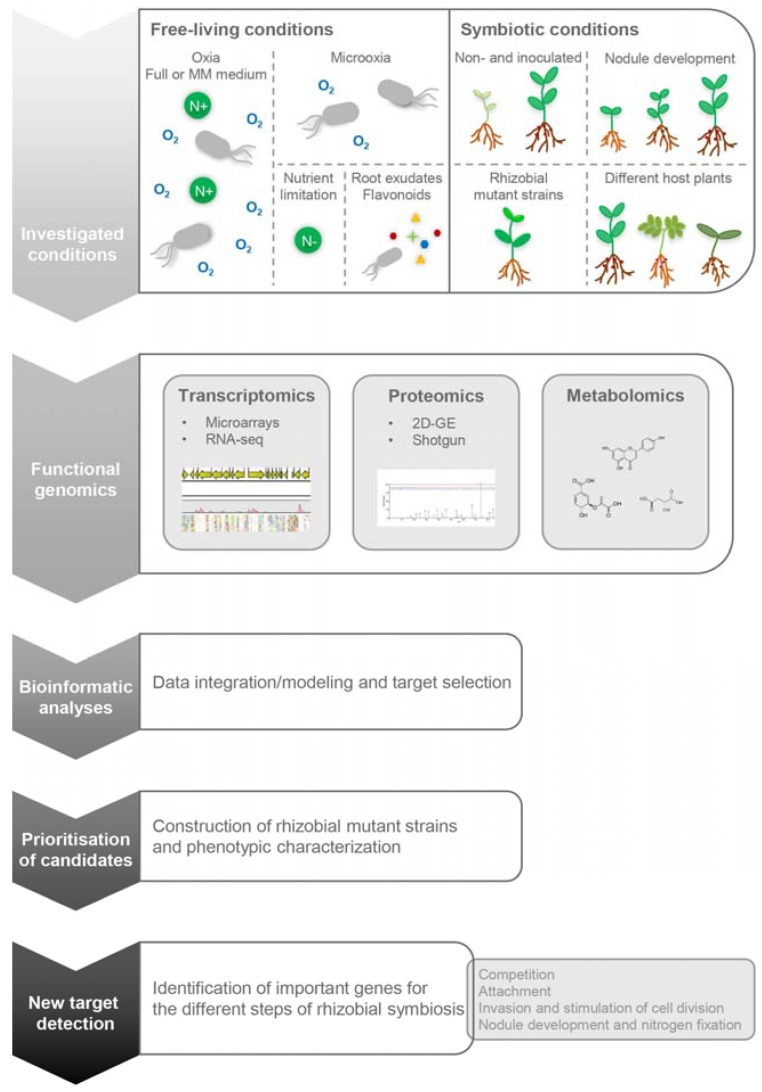
Workflow summarizing the main steps required for the characterization of genes important for different stages of the establishment of a functional symbiosis using functional genomics approaches. First, the investigated growth conditions are shown (**first chevron**), then the functional genomics technologies (**second chevron**) and their integrative data analyses (**third chevron**) used to prioritize candidate genes (**fourth chevron**). Finally, genes important for the different steps of symbiosis are identified and validated (**fifth chevron**). MM: minimal medium; 2D-GE: 2-dimensional gel electrophoresis; RNA-seq: RNA-sequencing.

Over the past few decades, several functional genomics studies have been published on rhizobia-legume symbioses. Often, depending on the research field, the study has focused on either the legume or on the rhizobial partner. This review aims to present the relative merits of different technical approaches in functional genomics for identifying genes/proteins/metabolites relevant for the establishment of a functional symbiosis with an emphasis on the rhizobial partner (Figure 1). For the sake of clarity, we provide a greater coverage on the global analysis performed on a standard model for investigation of the *Rhizobium*-legume symbiosis: the interaction between *Bradyrhizobium diazoefficiens* and *Glycine max* (soybean).

## 2. Functional Genomics of Rhizobia-Legume Symbiosis

### 2.1. Transcriptomics

#### 2.1.1. Microarrays versus RNA-Sequencing

Transcriptome analysis aims to quantify the expression level of each gene encoded in a genome in response to defined changes. In recent decades, several tools have been developed that allow researchers to unravel global transcriptional changes. The two most commonly employed techniques are based on either the hybridisation of cDNAs (DNA microarrays) or on deep sequencing of cDNA (RNA sequencing) [35,36,37]. RNA sequencing (RNA-seq) was first published in 2009 [37] and has an increased resolution (single base pair) and specificity (low background noise), a higher dynamic range of expression levels (>4 orders of magnitude), and a lower requirement for input material (a few nanograms) when compared with microarrays [38,39]. Moreover, it offers the possibility of simultaneously looking at the expression profiles in both the plant and bacterial partners during symbiosis [40]. In contrast to microarrays, in which the ribosomal RNA (rRNA) does not hybridise to the chip as homologous probes are not present, in RNA-seq, the abundant rRNA is ideally removed. The rRNA can be removed either right after the isolation of total RNA using the Microbe Express™ kit (Ambion, Waltham, MA, USA) [41] or Ribo-Zero^TM^ rRNA removal kit (Bacteria) (Epicentre, Madison, WI, USA) [42], or during library preparation using the Insert Dependent Adaptor Cleavage (InDA-C) technology of NuGEN (NuGEN, San Carlos, CA, USA), which is based on specific amplification and cleavage of cDNA derived from rRNA [43]. The development of differential RNA-seq (dRNA-seq), which includes the sequencing of a library enriched in primary transcripts, enables the mapping of all transcriptional start sites in different growth conditions and the identification of alternative and novel transcripts, including small regulatory RNAs [44,45].

We describe here the rhizobial transcriptomic changes occurring (i) after the addition of root exudates or flavonoids to free-living cultures; (ii) in nitrogen-limited conditions; (iii) during microaerobiosis; and (iv) when bacteria are living as bacteroids inside nodules of different legumes and at different developmental stages (Figure 1, Table 1).

#### 2.1.2. Transcript Profiling of Alpha-Rhizobia

The secretion of root exudates (RE) containing flavonoids into the rhizosphere is the first step of the symbiotic dialogue between the host plant and the rhizobia. Several studies have focused on the transcriptome profile of rhizobia in response to the perception of a single flavonoid during free-living conditions (Table 1). Other studies have unravelled the transcriptomic changes in the presence of RE from host and non-host legumes, as well as non-legume plants (Table 1). The study of Ramachandran and colleagues on *Rhizobium leguminosarum* biovar *viciae* 3841 cultured in the rhizospheres of *Pisum sativum* (host legume), *Medicago sativa* (a non-host legume), and *Beta vulgaris* (a non-legume) revealed the induction of a common set of genes (e.g., the *dctA* gene responsible for C_4_-dicarboxylate transport and the *rmrA* gene encoding an efflux pump) [58]. As expected, the induction of the nodulation (*nod*) genes was observed following the addition of exudates from both legumes, but not with those of *B. vulgaris*. Recently, the effects of *G. max* RE on two *B. diazoefficiens* strains, 4534 and 4222, were analysed by transcriptomics [53], which showed that several genes coding for two-component systems (*nodW, phyR-σ^EcfG^*), bacterial chemotaxis (*cheA*), ATP-binding cassette (ABC) transport proteins, and indole-3-acetic acid (IAA) metabolism were upregulated in the more competitive *B. diazoefficiens* strain 4534. A recent publication of Jiménez-Guerrero and colleagues extensively reviewed the transcriptomic studies performed in alpha-rhizobia with an emphasis on the effect of flavonoids on the activation of *nod* genes [98]. 

To elucidate the hierarchical regulatory cascade controlling *nif* (nitrogen fixation) transcription in the alpha-rhizobial strains *Sinorhizobium meliloti* and *B. diazoefficiens*, transcriptomic experiments using the wild type and different regulatory mutants have been performed under microoxic growth conditions (mimicking the environment inside nodules) and during symbiosis in several studies (Table 1) [46,48,50,55,62,63,67]. In 2004, the pioneer work of Barnett and colleagues investigated symbiotic gene expression using a dual-genome microarray that allowed the simultaneous examination of changes in the expression of both the bacterial and plant partners [66]. In a number of studies, the expression of certain gene clusters required for symbiotic functions—such as *fix* (respiration), *nif*, and *hup* (hydrogen uptake)—was shown to be activated under low-oxygen conditions and to be dependent on the sigma factor σ^54^ (or RpoN) [46,54,55,66]. However, the overlap between genes induced in microoxia and in symbiosis was partial and suggested that low oxygen is not the only signal required for an efficient lifestyle inside nodules. Unsurprisingly, most of the genes expressed differentially during symbiosis were downregulated, indicating that rhizobia invest a large proportion of their energy sources into reducing nitrogen for the host plant. Among the downregulated genes, some were involved in cell division, flagella synthesis, DNA and RNA metabolism, chemotaxis, phosphorus uptake and utilisation, glycolysis, and aerobic respiration [42,55,62,63]. In transcriptome studies analysing different nodule developmental stages, distinctive gene expression profiles were observed in early and mature bacteroids offering reference markers for bacteroid development [46,57,62,63,64,69]. In *B. diazoefficiens*-determinate *G. max* nodules, genes encoding the type 3 secretion system (T3SS) are induced in young nodules (10–13 days post infection, or dpi), while several transporter encoding genes (e.g., those for the transport of sulfate and sulfonate) are specifically upregulated in mature nodules (21–31 dpi) [51]. However, the expression of *nif* and *fix* genes was shown to be induced even in young bacteroids [46]. The outstanding work of Roux and colleagues coupled RNA-seq to laser-capture microdissection of specific *M. truncatula* nodule regions infected with *S. meliloti* to analyse plant and rhizobial gene expression profiles during the development of indeterminate nodules [69]. This study found that the expression of the sigma factor RpoN increased gradually and was maximal in terminally differentiated bacteroids (zone ZIII). Unexpectedly, the genes involved in the tricarboxylic acid (TCA) cycle showed downregulation in ZIII, suggesting a decline in nitrogen fixation in ZIII. Rhizobial adaptation to different plant hosts has been investigated using transcriptomics for the symbiosis of *B. diazoefficiens* with three different legumes: *Macroptilium atropurpureum*, *G. max*, and *Vigna unguiculata* [52] (discussion in Section 3), and in *Sinorhizobium fredii* NGR234 in symbiosis with *V. unguiculata* (determinate nodules) and *Leucaena leucocephala* (indeterminate nodules) [42]. Finally, transcriptome analyses based on dense tiling microarrays or RNA-seq allowed the confirmation of predicted small RNAs [99,100,101,102] and the identification of novel small RNAs upregulated and/or important during symbiosis [44,103,104].

#### 2.1.3. Transcript Profiling of Beta-Rhizobia

In contrast to alpha-rhizobia, only a few transcriptomic studies have been performed on beta-rhizobial symbioses. A comparative transcriptomic profiling analysis of two beta-rhizobia—*Paraburkholderia phymatum* STM815 and *Cupriavidus taiwanensis* LMG19424—as well as an alpha-rhizobium—*Rhizobium mesoamericanum* STM3625—in the presence of *M. pudica* RE [41] partly supported the previously observed differences in the competitive ability of these three strains to infect *M. pudica* [105]. Major changes were observed by RNA-seq in the highly competitive and original mimosa symbiont *P. phymatum* in response to mimosa RE. *P. phymatum* was shown to upregulate several genes involved in plant-bacterial interactions, such as *acdS* coding for a 1-aminocyclopropane-1-carboxylate (ACC) deaminase, an operon potentially involved in rhizobitoxine biosynthesis, a indole-3-acetic acid (IAA) biosynthesis gene, and clusters for secretion systems (a type 4 and a type 6 secretion system (T4SS and T6SS)). As expected, all three rhizobia reacted to RE. In addition to a common upregulation of *nod* genes, all three rhizobia reacted to RE with the induction of a newly identified fatty acid hydroxylase that may play a role during plant infection. Our own investigation on transcript profiling of a *P. phymatum* strain inside the root nodules of the promiscuous legume *Phaseolus vulgaris* identified an operon encoding a putative cytochrome o ubiquinol oxidase potentially needed for respiration inside the nodule as being highly upregulated during symbiosis [43]. In the same study, a transcript analysis was carried out on free-living *P. phymatum* growing under nitrogen-limited conditions that partially mimicked the environment encountered by rhizobia in nitrogen-deprived soils. Besides genes known to be involved in nitrogen assimilation, such as the key regulatory gene in control of nitrogen metabolism *ntrC, amtB* (encoding an ammonium transporter), and the *ure* cluster (encoding a urease), genes associated with important traits for legume infection, such as exopolysaccharide (EPS) production and motility, were upregulated [43]. Moreover, a recent comparative transcriptome analysis between *P. vulgaris* nodules induced by *P. phymatum* wild-type (Fix^+^) and by an *rpoN* mutant (Fix^−^) strain has confirmed the importance of RpoN in controlling the expression of *nif* genes and identified potential additional target genes of this alternative sigma factor in nodules [70]. 

### 2.2. Proteomics

#### 2.2.1. 2-Dimensional Gel Electrophoresis versus Liquid Chromatography Combined with Tandem Mass Spectrometry

Proteomics technologies have allowed for the investigation of expression changes directly at the protein level—that is, the players that ultimately carry out most functions in a living cell [106,107]. This ability gives a considerable advantage over transcriptomic studies. In addition, using the open software PeptideClassifier [108], the large majority of peptides can specifically be assigned to proteins originating from either the rhizobium or the host plant [73], which overcomes the problem caused by cross-hybridisation issues in microarrays [46]. Proteomics also opens avenues for analysing the subcellular localisation of proteins [109], discovering posttranslational modifications (PTMs) [90], studying their interaction partners [107], or identifying as-yet-unannotated protein coding genes [44,110,111]. However, it is more difficult to achieve good coverage of the expressed proteins, particularly for proteins expressed at a low abundance [112]. In addition, the data analysis poses greater challenges, as fewer standardised analysis software solutions are available when compared with transcriptomics [113]. Until the late 1990s, the identification of proteins was primarily performed using 2-dimensional gel electrophoresis (2D-GE)-based methods [114]. However, this has been changed with the introduction of high-performance liquid chromatography combined with tandem mass spectrometry (LC-MS/MS), also known as shotgun proteomics. In contrast to 2D-GE methodologies, shotgun proteomics is more sensitive and allows for the detection of hydrophobic membrane proteins [115]. Thus far, to our knowledge, proteomic approaches have been applied only to the study of alpha-rhizobial nitrogen fixing legume symbioses (Table 1) [116]. To identify proteins of interest, the protein expression profiles of free-living rhizobia are usually compared to the proteomes of bacteroids, or to those of cultures incubated with RE or specific flavonoids (Table 1).

#### 2.2.2. Protein Profiling of Free-Living Alpha-Rhizobia

Liu and coworkers [77] compared the proteome of two *B. diazoefficiens* strains showing different competitive abilities (strains 4534 and 4222) in response to *G. max* exudates and showed that the more competitive strain (4534) expressed more proteins potentially important for successful plant colonisation (e.g., regulation of signal transduction, chemotaxis, phytohormone metabolism, and ABC transporters). Several studies have investigated the extra- or intracellular proteomes of flavonoid-induced *B. diazoefficiens*, *R. leguminosarum* biovar *viciae*, and *R. etli* [74,75,78,82,83,84,85]. Proteomics on *B. diazoefficiens* cells treated with genistein allowed for the identification of proteins transported by T3SS [74,75]. In *R. etli*, several naringenin-induced exoproteins are involved in cell wall metabolism, EPS biosynthesis, and *myo*-inositol catabolic pathway [82]. Indeed, EPS and *myo*-inositol catabolism have been previously shown to be required for nodulation competitiveness [117,118].

#### 2.2.3. Protein Profiling of Alpha-Rhizobia Living Inside Nodules

The rhizobial protein profile in root and stem nodules of legumes has been investigated in several nitrogen-fixing symbioses. Using 2D-GE approaches, the first proteomic studies were performed on *G. max*–*B. diazoefficiens* nodules [119,120] and *M. truncatula* and *M. alba* nodules induced by *S. meliloti* [86,87,88]. These studies showed that the metabolic shift rhizobia undergo when entering symbiosis was reflected in the upregulation of proteins involved in N_2_ fixation, a subset of ABC-type transporters for transporting amino acids and inorganic ions (PO_4_ and Fe), and stress-related proteins such as chaperones, heat-shock proteins, and catalases.

A 2D-GE-based comparative proteomic investigation of free-living and bacteroid-state *B. diazoefficiens* suggested increased nitrogen metabolism and decreased nucleotide and fatty acid metabolism in bacteroids [72]. Thanks to the application of a more sensitive LC-MS/MS shotgun proteomic approach, the number of identified proteins inside *B. diazoefficiens* bacteroids increased substantially (from a few hundred to 2315). This approach also allowed for the identification of additional proteins involved in carbon and nitrogen metabolism (including a full set of TCA enzymes and enzymes involved in gluconeogenesis and the pentose phosphate pathway) and several proteins that were previously considered to be not expressed during symbiosis (e.g., in pathways for nucleoside and nucleotide biosynthesis) [73]. In another study based on the *B. diazoefficiens*–*G. max* symbiosis, proteins related to transcription, translation, protein folding, and degradation were shown to be upregulated in young nodules (7 and 10 dpi), while Nif and Fix proteins were upregulated in mature nodules [121]. An interesting investigation of proteins from root and stem nodules induced by a photosynthetic *Bradyrhizobium* sp. ORS278 in symbiosis with the semiaquatic plant *Aeschynomene indica* found a high correlation in the proteomes of the two types of nodules and discovered an important role for electron transfer flavoprotein FixA during these symbioses [79]. In contrast, proteins associated with the phototrophic ability of *Bradyrhizobium* sp. ORS278 were found to be expressed exclusively inside stem nodules. Proteome analyses of *Mesorhizobium loti* bacteroids isolated at different time stages during *Lotus japonicus* development (14, 21, and 28 dpi) revealed that bacteroids were nitrogen-deficient during their initial stages [81]. An exemplary large-scale, quantitative proteomic analysis was recently conducted on the model legume *M. truncatula* and its prokaryotic endosymbiont *S. meliloti*, which provided unique insights into PTMs of the nodule proteome and mechanisms regulating symbiosis [90]. With affinity enrichment technologies, Marx and colleagues [90] were able to identify the phosphorylation of rhizobial proteins associated with nitrogen fixation, such as NifH, NifX, and ferredoxin III, as well as the acetylation of NifH, NifB, FixT, and FixX. In the same study, 252 nodule-specific cysteine-rich peptides (NCRs) were detected and quantified over different nodule developmental stages. To identify rhizobial NCR targets, the abundances of the 3334 detected *S. meliloti* proteins were analysed and compared in young (10 dpi) and old (28 dpi) nodules. This comparison led to the identification of proteins related to regulation of cell cycle and cell division, transcriptional regulators, thereby partially confirming prior studies on rhizobial NCR targets [122,123].

### 2.3. Metabolomics

#### 2.3.1. Nuclear Magnetic Resonance versus Mass Spectrometry

Metabolomics aims to measure the presence and abundance of small molecules (metabolites) present within a system, the levels of which are constantly influenced by metabolic fluxes and enzyme activity [124]. The main reason for measuring metabolites is that they are more representative of metabolic phenotypes. Moreover, metabolome profiling requires relatively low material input and simple sample preparation [125]. Mass spectrometry (MS) and nuclear magnetic resonance (NMR) spectrometry are the two principal analytical approaches employed for metabolomic analysis [126]. Despite the fact that, nowadays, the majority of metabolomic studies are based on MS, both analytical methods have specific merits and drawbacks. NMR is superior in structural elucidation and can discriminate between molecules with the same mass, and its quantification of compounds in complex mixtures is very precise [127]. Furthermore, NMR offers the possibility of tracking metabolite dynamics intracellularly and in vivo [128]. On the other hand, MS is generally more sensitive and enables the simultaneous distinction of more metabolites, up to thousands of features with high-resolution instruments such as time-of-flight (TOF) or Orbitrap [125,129]. Both techniques can be coupled with chromatography-based systems, mainly gas chromatography (GC) and liquid chromatography (LC) [130]. With MS, chromatography is mainly employed to separate compounds with identical molecular weights or to reduce matrix effects (i.e., the interference between analytes that biases quantification). Metabolomic experiments can be roughly divided into two complementary approaches. The decision on which approach to pursue depends on the research purpose. Targeted metabolomics focuses on the measurement of a defined and typically small number of chemically characterised small molecules to obtain quantitative data. Untargeted metabolomics, in contrast, aims to comprehensively and nonselectively analyse the possibly well-known and possibly unknown low-molecular-weight molecules contained in a sample [131]. Untargeted metabolomics is a qualitative measurement that is particularly useful for the discovery of new metabolites [131]. 

#### 2.3.2. Metabolic Profiling of Nodules Induced by Alpha-Rhizobia

When looking at the interactions between two organisms—in this case, the rhizobial–legume symbiosis—the main disadvantage of metabolomics is that the origin of the metabolites often cannot be distinguished (i.e., whether they originated from the bacterial or the plant partner). Thus far, a few studies have been conducted using MS and NMR as analytical approaches to unravelling metabolic changes in the rhizobia–legume symbiosis by (i) comparing the metabolite profiles of root nodules and roots; (ii) performing kinetic experiments for nodule development; (iii) comparing nodules induced by the wild-type strains versus mutant strains; and (iv) analysing the metabolome of nodules from different plants infected by the same rhizobial strain. In several cases, the characteristic metabolites for a specific condition have been identified. Metabolic profiling of nodules and roots from four different *B. diazoefficiens* host plants, such as *G. max*, *V. unguiculata*, *V. radiata*, and *M. atropurpureum*, indicated that the amount of C_4_-dicarboxylate compounds and of several amino acids (glutamate, glutamine, proline, serine, and glycine) increased in all of the *B. diazoefficiens*-induced nodules [51]. Interestingly, the same set of metabolites also accumulated in nodules induced by *M. loti* in *L. japonicus* and by *S. meliloti* in *Medicago* spp., suggesting a role during symbiosis [94,96,97]. However, for each *B. diazoefficiens* host plant, a cluster of host-specific accumulated metabolites were identified; for example, ribose in *G. max*, tartaric acid in *V. radiata*, hydroxybutanoyloxybutanoate in *M. atropurpureum*, and catechol in *V. unguiculata*. In the same study [51], a metabolite analysis during different stages of nodule development revealed a maximum of C_4_-dicarboxylic acids in young nodules (13 dpi) and an accumulation of trehalose-phosphate and indole-3-acetate at 21 and 31 dpi, respectively. The accumulation of trehalose was also reported in nodules at 28–32 dpi in the same model (*B. diazoefficiens*–*G. max*) by another group [93]. In a study of the response of *G. max* root hairs to *B. diazoefficiens* inoculation, several (iso)flavonoids, amino acids, fatty acids, and carboxylic acids, as well as trehalose, were more abundant in root hairs following inoculation [92]. Metabolomic analysis of a rhizobial mutant defective in trehalose biosynthesis (Δ*otsA* Δ*treS* Δ*treY* triple mutant) suggested that *B. diazoefficiens* perceives osmotic stress during the earliest stages of the infection process and that trehalose may have a role in how nodulated roots cope with drought and other stress factors [92]. Metabolome profiling of *M. sativa* nodules induced by a *S. meliloti exoY* mutant (EPS^−^, Fix^−^) and a nitrogenase *nifH* mutant (Fix^−^) showed altered levels of metabolites involved in nitrogen and carbon metabolism, which are important for the establishment a functional symbiosis [96]. While nodules infected by an *exoY* mutant showed elevated levels of carbohydrates, the amount of all detected TCA intermediates was reduced. However, only the abundance of the two C_4_ -dicarboxylic acids, fumaric acid, and malic acid were found to be significantly reduced in nodules induced by the *S. meliloti nifH* mutant, which lack the nitrogenase and thus cannot provide ammonium to the host plant [96].

#### 2.3.3. Metabolic Profiling of Nodules Induced by Beta-Rhizobia

Recently, the first metabolomic study of the beta-rhizobia *P. phymatum* during symbiosis with *P. vulgaris* was published [70]; it showed that nodules formed by beta-rhizobia also accumulated compounds such as glutamine, chorismate, and arginine, when compared with the roots. In the same study, *P. vulgaris* nodules infected by the wild type (Fix^+^) and by the Fix^−^ *rpoN* mutant strain showed the accumulation of flavonoids in the nodules elicited by the Fix^−^ strain. In contrast, the amount of the precursor of the aromatic amino acids chorismate, and the level of other compounds such as alanine and ectoine, was decreased in Fix^−^ nodules. A preliminary comparison between the metabolomes of nodules induced by either alpha- or beta-rhizobia revealed that the C_4_-dicarboxylate compounds—succinate, malate, and fumarate—and the amino acid glutamine are more abundant in all nodules, when compared with roots. However, in contrast to nodules induced by alpha-rhizobia, certain compounds, such as glutamate, did not accumulate in beta-rhizobia-elicited nodules.

## 3. Integration of Different Omics Technologies

The integration of different functional approaches is a very powerful way to gain a better understanding of the cellular activities of rhizobia, and to elucidate how they can adapt their life styles from free-living to a symbiotic state. An integrative approach allows one to confirm expression data with metabolite-level information and to complement the results with an additional layer of knowledge. For example, for *B. diazoefficiens* bacteroids, transcriptomic data based on hybridisation of nodule cDNA on tiling microarrays [46,132] were integrated with proteomic results obtained from proteins isolated from bacteroids. The resulting final reference dataset for expression in *B. diazoefficiens* bacteroids contained genes detected only at the transcript level (e.g., those encoding several integral membranes or secreting proteins and weakly expressed genes such as transcriptional regulators) and proteins that were only identified by the proteomic approach [73]. Another study combined transcriptomics and proteomics to understand how *B. diazoefficiens* adapts to symbiotic life in three different host plants—*G. max*, *M. atropurpureum*, and *V. unguiculata* [52]. Among the genes and proteins specifically induced in one of the three host plants, a *B. diazoefficiens* gene cluster for a predicted ABC-type transporter was identified as *M. atropurpureum*-specific. Indeed, a strain in which the gene encoding this ABC transporter was mutated showed a symbiotic defect only in *M. atropurpureum*, but not in the two other legumes that were tested [52]. More recently, transcriptomic and proteomic studies of this model system (*B. diazoefficiens* in different host plants) were complemented by a metabolomic approach that confirmed the specific upregulation of certain pathways in a specific host [51]. For example, *G. max*’s specific upregulation of a threonine synthase at the transcript and protein levels was reflected by a specific accumulation of threonine metabolite in *G. max* nodules. In the same study, the identification of oxalate as a marker metabolite in young soybean nodules was in line with high expression levels of genes and enzymes responsible for the oxidation of oxalate to formate and CO_2_ in mature nodules [73]. *G. max* nodules induced by *B. diazoefficiens* mutants defective in nitrogen fixation *(nifA* and *nifH* mutant strains) were investigated by metabolomics in combination with transcriptomics [51]. Consistent with the fact that plants infected with both mutants phenotypically showed signs of nitrogen starvation, the amount of amino acids and their precursors was drastically reduced in the nodules induced by those mutants. Interestingly, in *G. max* nodules induced by the *B. diazoefficiens nifA* mutant strain, the amount of a phytoalexin was highly increased and, concomitantly, genes coding for T3SS were upregulated in *nifA* nodules, supporting the hypothesis that the legume induces a defence and stress response against this specific mutant. The regulons of NifA in *R. etli* growing in free-living conditions and during symbiosis with *P. vulgaris* were characterised using transcriptomics and proteomics. The study showed that besides *nif* and *fix* genes, a cytochrome monooxygenase operon and a putative hydroperoxide reductase were also expressed in a NifA-dependent way [56]. Thus far, to our knowledge, only one study has integrated different -omics technologies for studying beta-rhizobial symbiosis. Therein, transcriptomics and metabolomics were applied to shed light on the role of the key regulator of symbiosis *P. phymatum* RpoN in *P. vulgaris* nodules [70]. In several cases, metabolite measurements in Fix^+^ (wild type) and Fix^−^ (*rpoN* mutant) nodules confirmed transcript changes observed by RNA-seq analysis. For example, the increased amounts of aconitate and isocitrate were supported by the upregulation of the citrate synthase gene (*gltA*) in nodules induced by the *rpoN* mutant. The decreased amount of D-alanine/D-alanine in Fix^−^ nodules was in line with the downregulation of the D-alanine/D-alanine ligase and of the *mur* gene cluster required for peptidoglycan biosynthesis. It seemed that nodules elicited by the *rpoN* mutant strain accumulated a substantial amount of flavonoids, which have been proposed in another study to modify the rhizobial cell wall [82]. The accumulation of flavonoids in Fix^−^ nodules also suggested that, similarly to the situation in *B. diazoefficiens*, the non-fixing mutant strain generated a plant defence reaction. This was in line with the increased expression of resistance-nodulation-division (RND) efflux transporter genes and other genes associated with stress resistance inside nodules induced by the *rpoN* mutant strain.

For the *B. diazoefficiens*–*G. max* symbiosis, dRNA-seq data have been combined with proteomic and genomic analyses in a so-called proteogenomics approach to identify new open reading frames (ORFs) and correct existing genome annotations [44]. The new approach led to the discovery of 107 new *B. diazoefficiens* proteins and the novel N-terminus of 178 proteins in comparison to the existing annotation.

Finally, the integration and mapping of transcriptomic, proteomic, and metabolomic data onto known pathways in metabolic databases, such as KEGG (http://www.genome.jp/kegg/ http://pathways.embl.de/), MetaCyc, and BioCyc [133], facilitate data integration and identification of key differentially regulated pathways, and provide a basis for the prioritization and selection of candidate genes for further mutagenesis and validation experiments.

## 4. Future Perspective

Using functional genomics to elucidate the molecular processes underlying the establishment of an efficient nitrogen-fixing symbiosis between legumes and rhizobia is crucial for identifying the traits to increase the yield of agriculturally important crops. The rapid development of new, faster, and more sensitive instruments for measuring transcripts, proteins, and metabolites has allowed researchers to generate larger datasets from a smaller amount of input material. Metabolite profiling is a powerful way to monitor and assess the end products of bacterial and plant gene expression and can then be used to map these changes onto pathways and eventually identify new symbiosis-specific physiological changes. Metabolomics on nodules arrested at different developmental stages (*exoY* and *nifH* mutant strains) provide important insights that lead to a better understanding of nodule metabolism [51,96]. However, the fact that label-free metabolomic approaches do not allow differentiation between metabolites produced by the plant and those produced by the symbiont can be seen as a disadvantage of this technology. For this purpose, RNA-seq from nodules can be used. In fact, with the appropriate preparation of cDNA from the plant and bacterial partners, one can simultaneously monitor the transcript profile of both organisms in vivo. Therefore, the integration of -omics technologies is especially important for pinpointing important functions of each partner and providing a unified view of nodule metabolism. The combination of high-throughput approaches with innovative technologies such as laser-capture microdissection [69] enables researchers to record bacterial and plant gene expression in different stages of symbiotic interaction. Other innovative approaches such as proteogenomics, which allows for the identification of expression evidence for novel protein coding genes [44,110,111] and transposon sequencing (Tn-Seq) [134], could be employed to identify novel, as-yet-unannotated protein-coding genes or essential rhizobial genes required under symbiotic conditions. The complete sequencing of more rhizobial genomes and subsequent comparative genomic studies will help to reach an accurate list of the core, accessory, and unique genes and, lastly, will lead to the elucidation of the genetic elements required from both the bacterial and the plant partner for an efficient symbiosis. By generating and integrating -omics data for additional beta-rhizobial symbioses and comparing them with the existing data on alpha-rhizobial symbioses, we expect to identify mechanistic differences between alpha- and beta-rhizobial symbiosis, which was shaped by 50 million years separate evolution. Finally, further efforts from the scientific community are needed to develop platforms to store large -omics datasets in order to enable their integrative analysis and mining, and comparison between different rhizobial–legume model systems.

## Figures and Tables

**Table 1:** Summary of studies performed on rhizobial-legume symbioses using transcriptomics, proteomics, or metabolomics. For transcriptomic and proteomic studies, we have focused on studies of the microsymbiont.

Bacteria	Plant Host	Strain	Conditions	Reference
Transcriptomics (microarrays and RNA-seq *)				
**Alpha-rhizobia**				
*Bradyrhizobium diazoefficiens* USDA110	*Glycine max*	wt, *rpoN* double mt	microoxia (0.5% O_2_), nodule development (10, 13, 21 and 31 dpi)	[46]
*Bradyrhizobium diazoefficiens* USDA110	*G. max*	wt	bacteroids (28 dpi), salt stress	[47]
*Bradyrhizobium diazoefficiens* USDA110	*G. max*	wt	nodules (21 dpi)	[44] *
*Bradyrhizobium diazoefficiens* USDA110	*G. max*	wt, *regR* mt	microoxia (0.5% O_2_), nodule development (13 and 21 dpi)	[48]
*Bradyrhizobium diazoefficiens* USDA110		wt, *nodW* mt, *nodW-nswA* double mt with over-expression of *nwsB*	application of genistein	[49]
*Bradyrhizobium diazoefficiens* USDA110	*G. max*	wt, *fixk_2_* mt, *fixJ* mt	nodules (21 dpi)	[50]
*Bradyrhizobium diazoefficiens* USDA110	*G. max*	wt, *nifH* mt, *nifA* mt	nodules (21 dpi)	[51]
*Bradyrhizobium diazoefficiens* USDA110	*G. max, Macroptilium atropurpureum, Vigna unguiculata*	wt	nodules (21 or 31 dpi [*M. atropurpureum*])	[52]
*Bradyrhizobium diazoefficiens* 4534, 4222		wt	application of root exudates	[53] *
*Mesorhizobium huakuii* 7653R	*Astragalus sinicus*	wt	bacteroids (32 dpi)	[54] *
*Mesorhizobium loti* MAFF303099	*Lotus japonicus*	wt	microoxia (1.5% O_2_), bacteroids (42 dpi)	[55]
*Rhizobium etli* CFN42	*Phaseolus vulgaris*	wt, *nifA* mt	microoxia (1% O_2_), nodules (11 dpi)	[56]
*Rhizobium leguminosarum* biovar *viciae* 3841	*Pisum sativum, Vicia cracca*	wt	bacteroids (28 dpi), bacteroid development (7, 15, and 21 dpi)	[57]
*Rhizobium leguminosarum* biovar *viciae* 3841	*P. sativum, Medicago sativa, Beta vulgaris*	wt	application of root exudates, rhizosphere	[58]
*Rhizobium mesoamericanum* STM3625	*Mimosa pudica*	wt	application of root exudates	[41] *
*Rhizobium tropici* CIAT 899		wt, *nodD_1_* mt, *nodD_2_* mt	application of apigenin, salt stress	[59,60] *
*Sinorhizobium fredii* HH103		wt, *nodD_1_* mt, *ttsI* mt	application of genistein	[61] *
*Sinorhizobium meliloti* 1021	*Medicago truncatula, M. sativa*	wt, *bacA* mt	application of luteolin, microoxia (<1 µM O_2_), nodule development (8 and 18 dpi for Fix^+^ nodules and 11 dpi for Fix^−^)	[62]
*Sinorhizobium meliloti* 1021	*M. sativa*	wt	microoxia (<1 µM O_2_), bacteroid (18–22 dpi)	[63]
*Sinorhizobium meliloti* 1021	*M. sativa*	wt, *bacA* mt	nodule development (5, 8, 11, 14, or 18 dpi)	[64]
*Sinorhizobium meliloti* 1021		wt, wt with over-expression of *nodD_1_*	application of luteolin	[65]
*Sinorhizobium meliloti* 1021	*M. truncatula*	wt, *nodD_123_* triple mt, *nodD_123_* triple mt over-expressing *nodD_1_* or *nodD_3_, rpoN* mt*, fixJ* mt	application of luteolin, nodules (33–35 dpi)	[66]
*Sinorhizobium meliloti* 1021	*M. sativa*	wt, *fixJ* mt, *nifA* mt, *fixK* mt, *nifH* mt	microoxia (2% O_2_), nodules (14 dpi)	[67]
*Sinorhizobium meliloti* 2011	*M. truncatula*	wt	nodules (10 dpi)	[68] *
*Sinorhizobium meliloti* 2011	*M. truncatula*	wt	nodule development (10 or 15 dpi, laser dissection)	[69] *
*Sinorhizobium* sp. NGR234	*V. unguiculata, Leucaena leucocephala*	wt	bacteroids (21 dpi or 31 dpi for *L. leucocephala*)	[42] *
**Beta-rhizobia**				
*Cupriavidus taiwanensis* LMG19424	*M. pudica*	wt	application of root exudates	[41] *
*Paraburkholderia phymatum* STM815	*P. vulgaris*	wt, *rpoN* mt	nodules (21 dpi)	[43,70] *
*Paraburkholderia phymatum* STM815	*M. pudica*	wt	application of root exudates	[41] *
*Paraburkholderia phymatum* STM815		wt	nitrogen starvation	[43] *
Proteomics (2-D GE and LC-MS/MS *)				
**Alpha-rhizobia**				
*Bradyrhizobium diazoefficiens* USDA110	*G. max*	wt	bacteroids (28 dpi or 21 dpi)	[71,72] [46,73] *
*Bradyrhizobium diazoefficiens* USDA110	*G. max, M. atropurpureum, V. unguiculata*	wt	bacteroids (21 or 31 dpi [*M. atropurpureum*])	[52] *
*Bradyrhizobium diazoefficiens* USDA110		wt**, flagellin*-ttsI* double mt, flagellin-T3SS double mt	application of genistein	[74]
*Bradyrhizobium diazoefficiens* USDA110		wt, flagellin mt	application of genistein	[75] *
*Bradyrhizobium diazoefficiens* USDA110		wt	microoxia (2% O_2_), anoxia	[76]
*Bradyrhizobium diazoefficiens* 4534, 4222		wt	application of root exudates	[77]
*Bradyrhizobium diazoefficiens* CPAC 15		wt	application of genistein	[78]
*Bradyrhizobium* sp. ORS278	*Aeschynomene indica*	wt	bacteroid development (14 and 21 dpi)	[79] *
*Mesorhizobium loti* MAFF303099	*L. japonicus*	wt	nodules (49 dpi)	[80] *
*Mesorhizobium loti* MAFF303099	*L. japonicus*	wt	bacteroid development (14, 21 and 28 dpi)	[81] *
*Rhizobium etli* CFN42	*P. vulgaris*	wt, *nifA* mt	bacteroids (11 dpi)	[56]
*Rhizobium etli* CE3		wt	application of naringenin	[82] *
*Rhizobium leguminosarum* bv *viciae* 3841		wt	application of naringenin	[83,84] *
*Rhizobium leguminosarum* bv *trifolii* ANU843		wt	application of 7,4′-dihydroxyflavone (DHF)	[85]
*Sinorhizobium meliloti* 1021	*M. truncatula, Melilotus alba*	wt	nodules, bacteroids (12 dpi)	[86,87,88]
*Sinorhizobium meliloti* 2011	*M. truncatula*	wt	bacteroids, nodule development (+ or − drought stress) (3 and 6 dpi)	[89] *
*Sinorhizobium meliloti*	*M. truncatula*	wt	bacteroids, nodule development (10, 14 and 28 dpi)	[90] *
*Sinorhizobium meliloti* 2011		wt, pRm211aΔ14-16, pRm2011a cured	application of luteolin	[91]
*Metabolomics*				
**Alpha-rhizobia**				
*Bradyrhizobium diazoefficiens* USDA110	*G. max, M. atropurpureum, V. unguiculata, Vigna radiata*	wt, *nifA* mt, *nifH* mt	nodule development (13, 21 and 31 dpi)	[51]
*Bradyrhizobium diazoefficiens* USDA110	*G. max*	wt	root hairs	[92]
*Bradyrhizobium diazoefficiens* USDA110	*G. max*	wt	bacteroids (28–32 dpi)	[93]
*Mesorhizobium loti* R7A	*L. japonicus*	wt	nodules (84 dpi)	[94,95]
*Sinorhizobium meliloti* 1021	*M. sativa*	wt, *exoY* mt, *nifH* mt	nodules (21 dpi)	[96]
*Sinorhizobium meliloti* 1021	*M. truncatula*	wt, *fixJ* mt	nodules (14 dpi)	[97]
**Beta-rhizobia**				
*Paraburkholderia phymatum* STM815	*P. vulgaris*	wt, *rpoN* mt	nodules (21 dpi)	[70]

* Indicates studies performed with RNA-seq (transcriptomics) or liquid chromatography combined with tandem mass spectrometry (LC-MS/MS) (proteomics); wt: wild-type strain; mt: mutant strain; dpi: days post infection.
